# Magnetic balloon–assisted percutaneous endoscopic jejunostomy

**DOI:** 10.1016/j.vgie.2026.02.001

**Published:** 2026-02-05

**Authors:** Wesley Wright, Matthew R. Krafft

**Affiliations:** Department of Gastroenterology & Hepatology, West Virginia University Medicine, Morgantown, West Virginia, USA

## Abstract

**Background and Aims:**

Magnetic balloon technology was created to assist in colonic loop reduction in cases of difficult or failed colonoscopy. It has since been recognized that the device can facilitate percutaneous endoscopic jejunostomy (PEJ) by readying a jejunal puncture point. This case describes magnetic balloon–assisted PEJ placement.

**Methods:**

A patient with obesity with prior Roux-en-Y gastric bypass required percutaneous enteral feeding tube placement. Percutaneous endoscopic gastrostomy insertion was not possible because of intrathoracic location of the gastric pouch. A jejunal puncture point was not identifiable using standard techniques because of central adiposity (eg, transillumination and 1-to-1 palpation).

**Results:**

A ferromagnetic balloon and permanent magnet were used to magnetically attract and anchor a loop of jejunal Roux limb to the anterior abdominal wall, thereby enabling identification of a jejunal puncture point using 1-to-1 palpation. A 20F “pull-type” enteral feeding tube was inserted into the jejunal Roux limb. The procedure was completed without fluoroscopy. No adverse events occurred.

**Conclusions:**

Historically, jejunal puncture point localization and/or trocar passage has been the rate-limiting step of PEJ, accounting for approximately 13% technical failure rate. Magnetic balloon technology may assist with transabdominal jejunal access by magnetically attracting the jejunum to the abdominal wall.

## Background

Standard techniques for jejunal puncture point localization during direct percutaneous endoscopic jejunostomy (PEJ) include transillumination and 1-to-1 palpation. Fluoroscopic guidance can be used when standard techniques fail. Although effective, fluoroscopy introduces radiation, increases procedure time, and demands specialized equipment and personnel. To address these limitations, a novel PEJ strategy has been trialed using magnetic balloon technology. Magnetic balloon anchoring was developed to assist in colonoscopy completion during difficult colonoscopy (looping).^1^^,^^2^ The magnetic balloon device can also be used off-label during PEJ to magnetically attract and anchor a loop of jejunum to the anterior abdominal wall, allowing a jejunal puncture point to be identified with standard techniques.

## Case

A 35-year-old female patient (body mass index = 30) with prior Roux-en-Y gastric bypass presented with acute quadriplegia secondary to a motor vehicle accident. Percutaneous enteral feeding access was requested by the primary team for projected nutritional support >4 weeks in the setting of oropharyngeal dysphagia. PEG insertion was not possible because of intrathoracic location of the gastric pouch; therefore, PEJ placement was selected.

**Figure 1:**
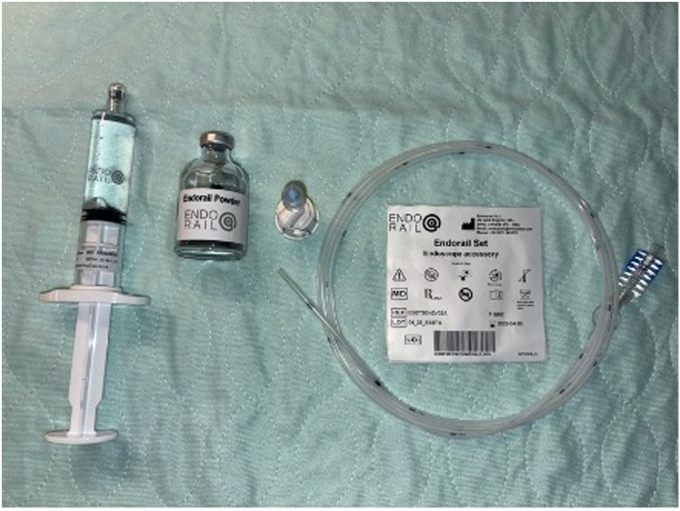
Magnetic balloon technology, accessory set: saline solution (23 mL of prefilled hypertonic solution); carbonyl iron microsphere powder (29 g); and balloon catheter (2.3- × 6-cm balloon dimensions once inflated with ferromagnetic fluid [powder + saline solution]).

**Figure 2:**
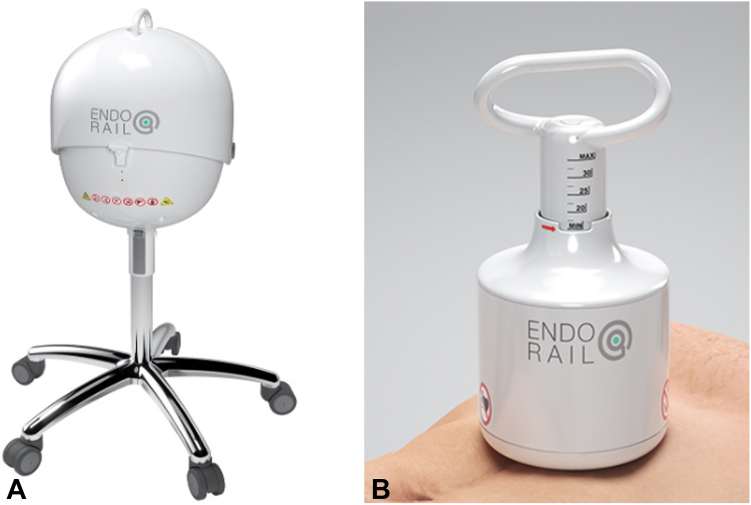
Cart (**A**) stores the permanent magnet handpiece (**B**). Magnetic power is adjusted by twisting the magnet handpiece handle clockwise (↑power) or counterclockwise (↓power).

The patient was placed in the supine position (Video 1, available online at www.videogie.org). A colonoscope was introduced through the mouth and advanced to the proximal jejunal Roux limb. Because of central adiposity, a jejunal puncture point could not be found using transillumination and/or 1-to-1 palpation. Magnetic balloon technology was used to localize a jejunal puncture point (Figs. 1 and 2).

**Figure 3:**
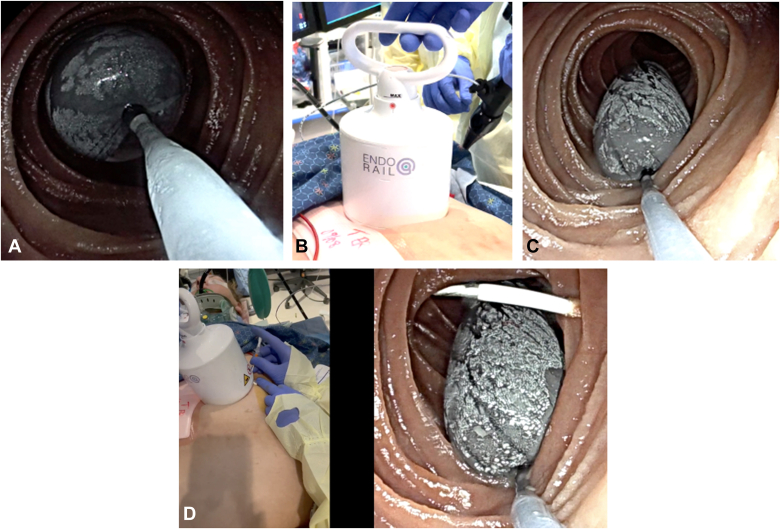
**A-D,** Identification of a jejunal puncture point using the magnetic balloon: (**A**) balloon catheter inflated with ferromagnetic fluid within the jejunum; (**B**) handpiece (permanent magnet) placement onto the overlying abdominal wall. The handpiece was adjusted to maximum power (handle twisted clockwise to lower the permanent magnet toward the abdominal wall); (**C**) after permanent magnet placement onto the overlying abdominal wall, iron powder within the balloon catheter arranged into magnetic field lines, parallel to the magnetic field (Zenith sign). The jejunal lumen was magnetically apposed (anchored) to the underside of the abdominal wall; (**D**) insertion of trocar needle with sheath into the magnetically apposed (stabilized) jejunum. The remainder of the PEJ procedure was completed using the pull-type technique.

The magnetic balloon catheter was passed through the colonoscope working channel and into the jejunal lumen. The assistant inflated the balloon catheter with ferromagnetic fluid (Fig. 3A). The permanent magnet handpiece was placed on the patient's anterior abdominal wall, near the distal tip of the colonoscope and ferromagnetic balloon. The handpiece was screwed clockwise to lower the permanent magnet toward the abdominal wall (Fig. 3B). The ferromagnetic balloon became magnetically anchored to the overlying abdominal wall/permanent magnet (Fig. 3C), allowing 1-to-1 palpation. Jejunopexy was created using 2 T-fasteners (Avanos Medical, Inc, Alpharetta, Ga, USA) to fix the jejunum to the abdominal wall. The trocar needle was passed underneath the handpiece into the jejunum (Fig. 3D). The permanent magnet was retracted from the abdominal wall by twisting the handpiece counterclockwise. The ferromagnetic balloon was deflated and extracted from the working channel of the colonoscope. The remainder of the PEJ procedure was completed using the pull-type technique.^3^ No adverse events occurred.

## Conclusion

Technical success of PEJ tube insertion is relatively low (87%), mainly because of challenges in jejunal puncture point localization (ie, jejunum has a narrow luminal diameter and a mobile mesenteric suspension).^4^^,^^5^ In this case, magnetic balloon technology facilitated PEJ creation in a patient with obesity, without fluoroscopy. Magnetic balloon technology attracted a loop of jejunal Roux limb to the anterior abdominal wall, thereby anchoring the jejunum to the abdominal wall for completion of PEJ. Further studies are needed to corroborate the utility of magnetic balloon technology for use in PEJ.

## Limitations

**Figure 4:**
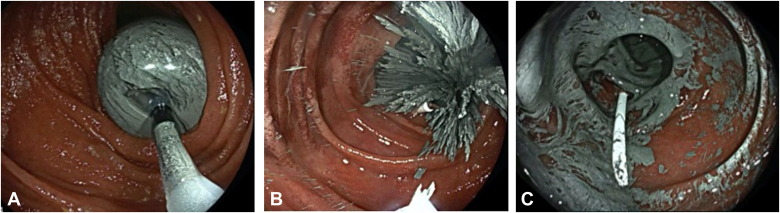
**A-C,** Sequential images of ferromagnetic balloon rupture during passage of transabdominal T-fastener (Avanos Medical, Inc, Alpharetta, Ga, USA) passage (jejunopexy) in the PEJ procedure. **A,** Inflated ferromagnetic balloon catheter within the jejunum magnetically apposed to the anterior abdominal wall (iron powder arranged in magnetic field lines). **B,** Inadvertent rupture of ferromagnetic balloon when struck by a sharp object (transabdominal T-fastener passage during jejunopexy). Iron-containing fluid is suspended mid-air in magnetic field lines because of the permanent magnet placement onto the overlying anterior abdominal wall. **C,** Iron-containing fluid in liquid nonmagnetized form after removal of the overlying permanent magnet.

Magnetic balloon technology carries some limitations:

• The magnetic balloon system includes a permanent magnet handpiece, which is contraindicated for both patients and staff with implanted ferromagnetic medical devices.
• No clinical or experimental data have yet determined whether organ (colonic) interposition risk is mitigated. It is recommended to continue practicing the established safety maneuvers before placing PEJ, including transillumination, digital indentation, and the safe-tract method.
• Body habitus can present limitations for balloon anchoring. Experiments have shown the magnetic balloon attraction to the overlying magnet handpiece is effective up to approximately 9 cm of distance. The presence of intervening connective tissues reduces the magnetic range to <9 cm.
• Ferromagnetic solution within the balloon contains 29 g of carbonyl iron microspheres. If unintentional balloon rupture occurs in the jejunum during PEJ (Fig. 4A-C), systemic absorption of carbonyl iron is improbable or negligible without gastric acid for iron solubilization.^6^ Data are limited regarding the risk of endoscope damage from suctioning ferromagnetic fluid in the event of balloon rupture. Current practice is to avoid suctioning ferromagnetic fluid if it does not impair endoscopic visualization. If suctioning occurs, prompt cleansing (water flushing) of the working channel is recommended.

## Disclosure

The authors disclosed no financial relationships.

## Supplementary data

Video 1 Demonstration of magnetic balloon–assisted percutaneous jejunostomy with inclusion of Disclosures, Disclaimer, Equipment used for the procedure, Background of the case being presented, Demonstration of the magnetic balloon technology, Demonstration of the magnetic balloon device components, Contraindications to the use of magnetic balloon technology, Magnetic balloon safety with demonstration of complication (balloon rupture), Deployment mechanism for jejunopexy with T-fasteners, Patient setup for procedure, Demonstration of the completed magnetic balloon–assisted PEJ, Conclusion, and Bibliography.
